# Conservation of Bacterial Lipopolysaccharide Binding by SARS-CoV-2 Spike across Major Viral Variants

**DOI:** 10.34133/csbj.0040

**Published:** 2026-04-02

**Authors:** Firdaus Samsudin, Ganna Petruk, Li Rui, Artur Schmidtchen, Peter J. Bond

**Affiliations:** ^1^ Bioinformatics Institute (BII), Agency for Science, Technology and Research (A*STAR), Singapore 138671, Singapore.; ^2^ Division of Dermatology and Venereology, Department of Clinical Sciences, Lund University, SE-22184 Lund, Sweden.; ^3^ Department of Dermatology, Skåne University Hospital, Lund, Sweden.; ^4^ Department of Biological Sciences, National University of Singapore, Singapore 117543, Singapore.

## Abstract

Severe acute respiratory syndrome coronavirus 2 (SARS-CoV-2) pathogenesis is shaped not only by viral entry mechanisms but also by interactions with host and microbial factors. The viral spike (S) protein can bind Gram-negative bacterial lipopolysaccharide (LPS), a driver of hyperinflammation in severe COVID-19. How viral evolution over the years alters this interaction remains unclear. Here, we investigated LPS binding across major SARS-CoV-2 variants that emerged over the course of the pandemic (2019–2023) from the ancestral Wuhan-Hu-1 strain to Omicron subvariants BA.1, XBB.1.5, and BA.2.86. Structural mapping revealed multiple mutations near a cryptic lipid-binding pocket in the receptor binding domain (RBD). Using extensive atomic-resolution molecular dynamics (MD) simulations with free energy calculations, validated by biochemical binding assays and fluorescence quenching experiments, we show that these mutations weaken binding to the lipid A component of LPS. However, full-length LPS binds with similar affinity to most variants likely due to increased positive electrostatic potential of the RBD, promoting compensatory interactions with negatively charged LPS inner core sugars. Together, these findings uncover an evolutionary balance that preserves S protein–LPS engagement through distinct molecular mechanisms, suggesting that emerging variants may retain the capacity to potentiate hyperinflammation during infection.

## Graphical Abstract

**Figure:**
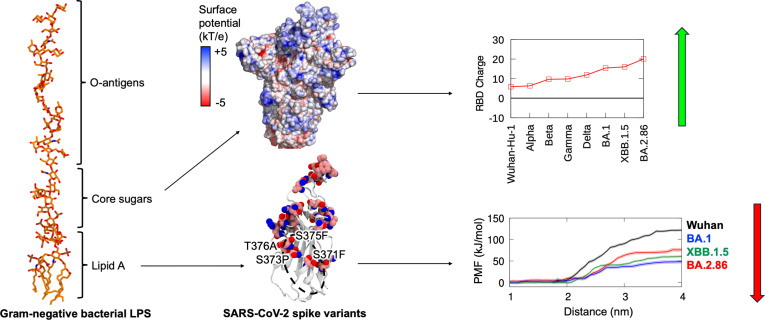

## Introduction

Since the emergence of COVID-19 in December 2019, the causative pathogen, severe acute respiratory syndrome coronavirus 2 (SARS-CoV-2), has continuously evolved from its ancestral Wuhan-Hu-1 variant. The emergence of adaptive mutations, particularly on the S protein, has been crucial in evading the host immune response [1]. While the first few key variants, including former variants of concern (VOCs) such as Alpha (B.1.1.7), Beta (B.1.351), Gamma (P.1), and Delta (B.1.617.2), gained around 10 mutations each, the first saltation variant, Omicron BA.1, had over 30 mutations on the S protein [2]. As the virus continues to evolve, several Omicron subvariants have been discovered including the XBB sublineages, such as the former variant of interest (VOI) XBB.1.5, which was the predominant circulating variant worldwide in 2023 [3]. Unexpectedly, a new reported saltation variant, BA.2.86, was identified in mid-August 2023 with more than 30 mutations relative to its parental BA.2 variant and over 50 mutations compared to the original Wuhan-Hu-1 variant, representing the biggest leap in SARS-CoV-2 evolution since BA.1 [4,5]. The variant was subsequently detected in several countries across Asia, Europe, Africa, and North America [6–8], suggesting a silent global spread. In April 2024, the successor of the BA.2.86 variant, designated JN.1, whose S protein has one additional mutation, became the most predominant variant worldwide [9,10]. Given the large number of mutations, the World Health Organization (WHO) then assigned both BA.2.86 and JN.1 as VOIs. As the S protein of SARS-CoV-2 continues to evolve, understanding how virulence differs between emerging variants to the ancestral Wuhan-Hu-1 strain is of utmost importance to predict the trajectory of the disease and prepare for potential future outbreaks.

One hallmark feature of the S protein is its ability to bind to Gram-negative bacterial lipopolysaccharide (LPS) and “boost” Toll-like receptor 4 (TLR4)-mediated proinflammatory responses [11,12]. Our previous study reported a nanomolar *K*_D_ constant between the Wuhan-Hu-1 S protein and *Escherichia coli* LPS, which is similar to that of LPS co-receptor, CD14, suggesting a physiological relevance for this interaction [11]. We then uncovered 3 distinct LPS binding sites on the S protein: 2 cryptic pockets in the S1 subunit, specifically in the N-terminal domain (NTD) and the receptor binding domain (RBD), which are known to bind small hydrophobic molecules from various structural studies [13–16], as well as one surface groove located between the protomers of the S protein trimer in the S2 subunit [12]. Binding assays and free energy calculations showed that the S1 pockets represent higher affinity sites with nanomolar *K*_D_ constants, whereas the S2 surface groove represents lower affinity site with micromolar *K*_D_ constant [12]. Given that S protein binds to LPS with a similar range of affinities to that of CD14 [11,12], we proposed that the S protein acts as an additional LPS carrier to sequester the lipids from free bacterial envelopes or aggregates, and transfers them to the LPS co-receptor CD14. This precedes delivery to the terminal receptor TLR4 in complex with its MD-2 co-receptor, which then elicits downstream inflammatory reactions. Patients with metabolic syndrome, which is associated with high blood level of LPS [17], have a higher risk to develop severe COVID-19 complications including systemic hyperinflammation [18,19]. Additionally, LPS binding triggers S protein aggregation, leading to the formation of amyloid structures [20], which could potentially cause neurodegeneration [21]. Our studies thus established a molecular basis for dysfunctional immune receptor activation and neuropathologies observed in COVID-19 patients via interaction between LPS and the S protein.

The effects of mutations in emerging variants on S protein–LPS interaction remain largely unclear. Our preliminary experiments and simulations demonstrated a moderate reduction in LPS boosting in vitro and in vivo for the Omicron BA.1 variant [12]. Three serine mutations (S371L, S373P, and S375F) were found around the LPS binding pocket on the RBD of the Omicron S protein. We hypothesized that these mutations abolished key interactions between the protein and lipid A (the primary bioactive lipid component of LPS), resulting in structural distortion of the gating helix that controls the opening and closing of the cryptic pocket, consequently reducing lipid A binding affinity compared to the Wuhan-Hu-1 variant. However, it is not known how the continuous evolution of the virus to the more recent variants affects LPS binding to the S protein.

In this study, we focus on S proteins from several landmark variants over the 4-year period of the pandemic (BA.1, XBB.1.5, and BA.2.86) to investigate how emerging mutations affect the binding of LPS. Specifically, our aim was to map key mutations with respect to the LPS binding sites, determine LPS binding affinities, and explore how the S protein adapts to retain its capacity for LPS binding despite various mutations. Collectively, our findings highlight the complex evolutionary consequences of SARS-CoV-2 S protein mutations on bacterial LPS interaction, with important implications for COVID-19 pathogenicity.

## Results

### Emerging variants display mutations around RBD cryptic pocket

**Fig. 1.**
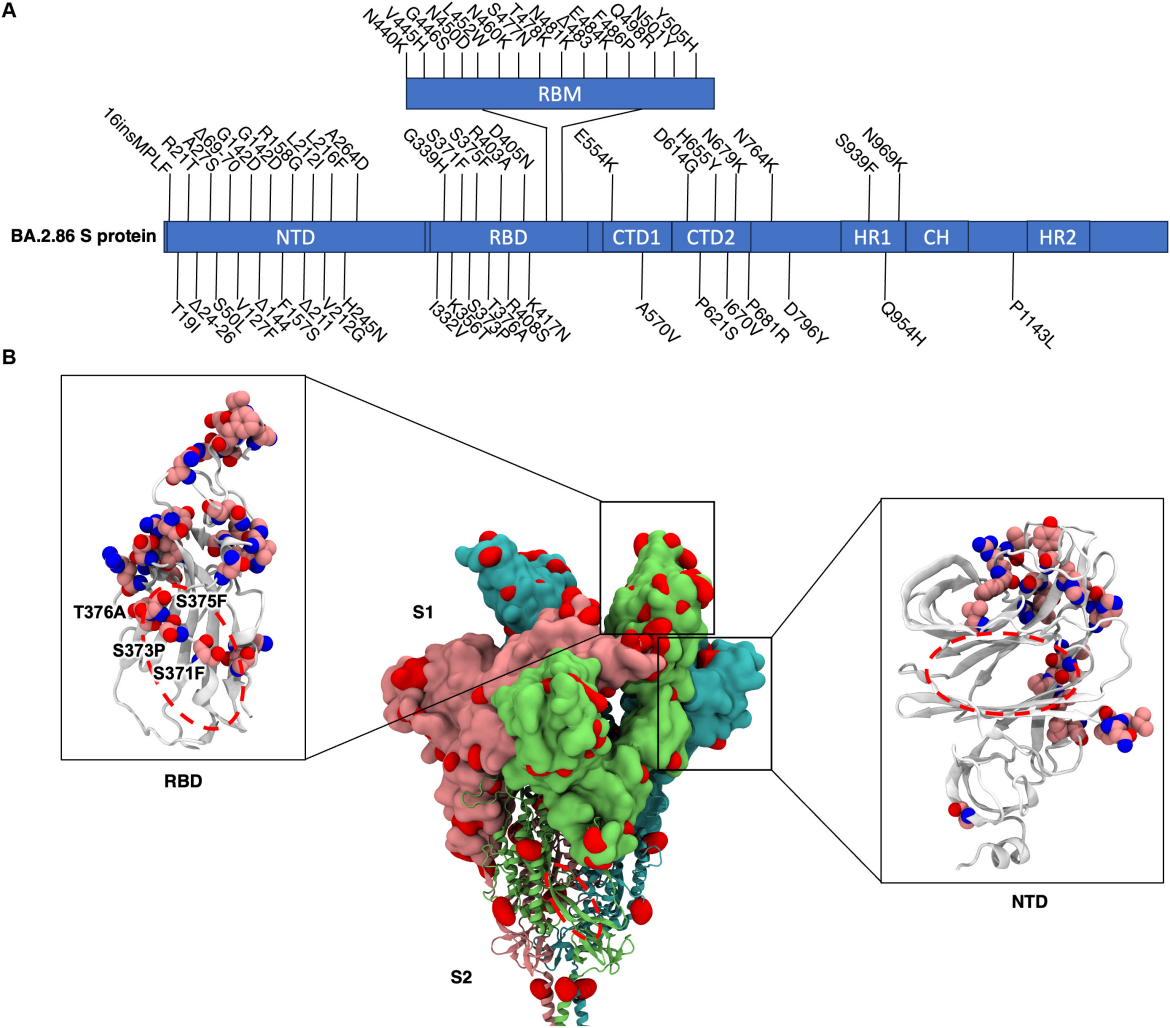
Mutations on the BA2.86 variant relative to LPS binding sites. (A) List of mutations on the S protein of SARS-CoV-2 BA.2.86 variant compared to the ancestral Wuhan-Hu-1 variant. NTD, N-terminal domain; RBD, receptor binding domain; RBM, receptor binding motif; CTD, C-terminal domain; HR, heptad repeat; CH, central helix. (B) Center: The positions of all mutations mapped onto the surface of the S protein ECD and colored red. The 3 chains of the S protein trimer are colored green, pink, and cyan. The S1 subunit is shown in surface representation, while the S2 subunit is shown in cartoon representation. The position of the LPS binding site on the S2 subunit is indicated by the dashed circle. Enlarged image, left: Mutations on the RBD shown in pink van der Waals representation. The position of the LPS binding site is indicated by a dashed circle, and the 4 nearby mutations are labeled. Enlarged image, right: Mutations on the NTD relative to the position of the LPS binding site.

We first performed a multiple sequence alignment of the S protein from the ancestral Wuhan-Hu-1 variant, all former VOCs (Alpha, Beta, Gamma, Delta, and Omicron BA.1), as well as former VOIs (XBB.1.5 and BA.2.86) (Fig. S1). The sequence data were obtained from https://covariants.org/variants enabled by the GISAID consortium [22]. As expected, most of the mutations occur within the S1 subunit, particularly the NTD and RBD, with the S2 subunit relatively well-conserved. For further analyses, we selected 3 key Omicron variants—BA.1, XBB.1.5, and BA.2.86—as they were the predominant variants at different time points during the COVID-19 pandemic and they all have available structural data in the Protein Data Bank (PDB). To elucidate the locations of all mutations found in the S protein of these variants relative to the LPS binding site, we mapped the positions of all mutated residues onto the cryo-electron microscopy (EM) structures of the S protein ectodomain (ECD) of each variant (Fig. 1 and Figs. S2 and S3). Most mutations are located in the S1 subunit, and no mutations were found in the vicinity of the S2 LPS binding groove. The BA.1, XBB.1.5, and BA.2.86 S1 subunits have accumulated 23, 33, and 45 mutations, respectively. In the NTD, almost all of the mutations are located in the antigenic supersite, the region at the outer edge of the domain, which is one of the primary epitopes of neutralizing antibodies [23,24]. Similarly, in the RBD, most mutations map to the receptor binding motif (RBM), which is also a vital antibody target. Noteworthy, the LPS binding site on the RBD has 3 mutations in BA.1 [S371L, S373P, and S375F (Fig. S2B)] and 4 mutations in BA.2.86 and XBB.1.5 [S371F, S373P, S375F, and T376A (Fig. 1B and Fig. S3B, respectively)]. These mutations map to the gating helix of the cryptic hydrophobic pocket on the RBD. In contrast, we did not find any mutations within the vicinity of the LPS binding site on the NTD. Our previous study also showed that RBD represents a high-affinity binding site for LPS [12]. Therefore, further analyses were focused on the RBD from these variants.

### LPS binding is conserved in emerging variants

Due to the mutations on the gating helix of the RBD cryptic pocket of these variants, we hypothesized that LPS binding might be compromised. To investigate whether the RBD from these variants can still bind to LPS, we performed blue native–polyacrylamide gel electrophoresis (BN-PAGE) followed by Western blotting. The purity of S protein RBDs was first evaluated using sodium dodecyl sulfate (SDS)–PAGE (Fig. S4). We found that all 4 RBDs from Wuhan-Hu-1, BA.1, XBB.1.5, and BA.2.86 migrated to around 45 kDa producing homogeneous bands under reducing conditions.

**Fig. 2.**
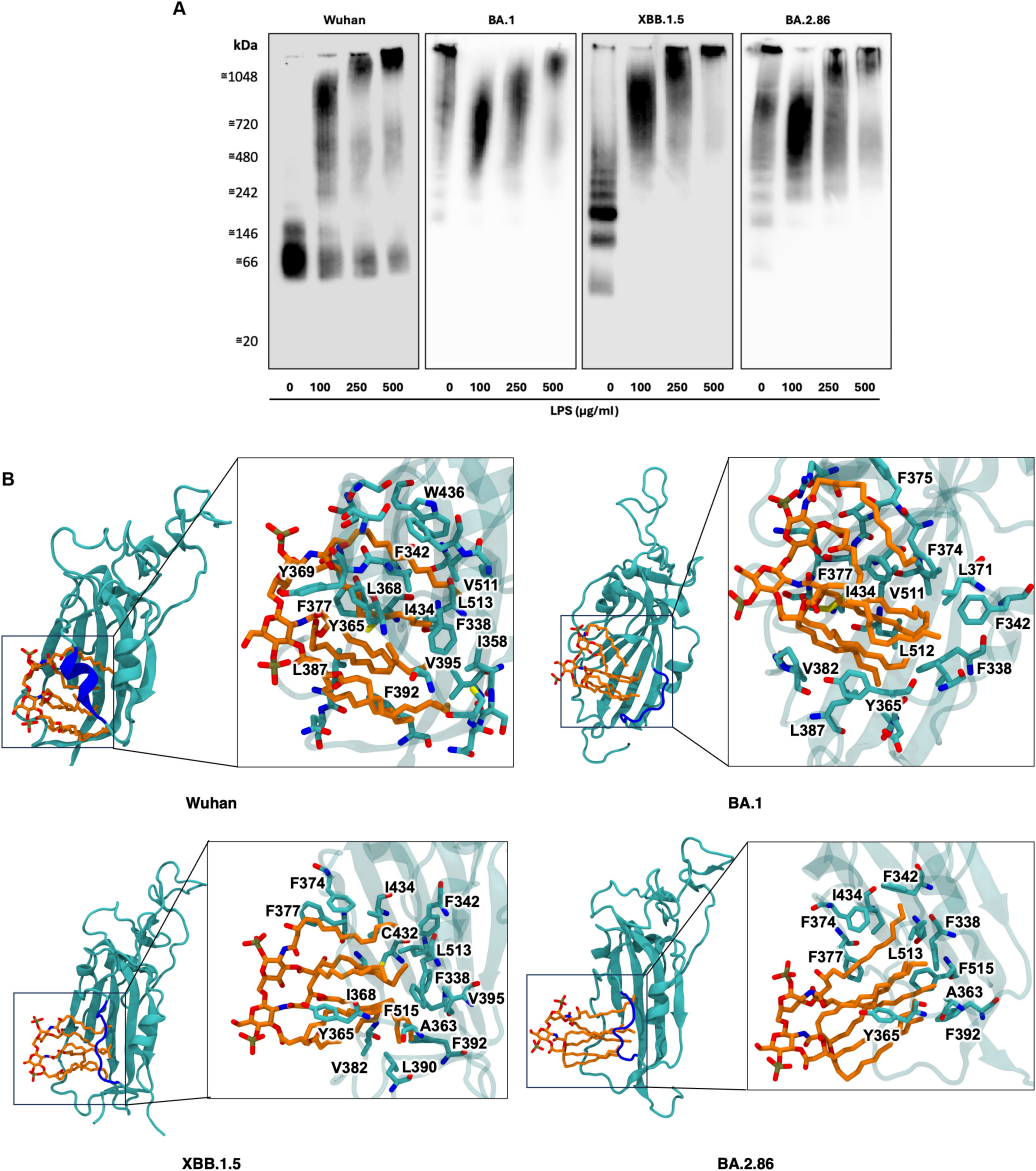
LPS binds to spike RBD from all variants. (A) Evaluation of LPS binding by SARS-CoV-2 S protein RBD and its mutants by BN-PAGE. SARS-CoV-2 S protein RBD and its mutants were incubated with 0 to 500 μg/ml LPS, separated by BN-PAGE and detected by Western blotting. One representative image of 3 independent experiments is shown (*n* = 3). (B) Three independent 1-μs simulations were performed for Wuhan-Hu-1, Omicron BA.1, Omicron XBB.1.5, and Omicron BA.2.86 S protein RBDs bound to lipid A. The figures show a representative structure obtained from clustering analysis of the concatenated trajectories using an RMSD cutoff of 0.35 nm. Lipid A is shown in orange and RBD in cyan with the gating helix (residues 364 to 371) shown in blue. Enlarged images show key residues involved in binding.

In BN-PAGE, there is a shift in migration for all 4 variants in the presence of *E. coli* LPS, confirming that each tested RBD interacts with LPS under native conditions (Fig. 2A). For the Wuhan-Hu-1 RBD alone, the main band was observed at approximately 66 kDa, with an additional faint band around 146 kDa, indicating that under native conditions the protein may exist in distinct oligomeric or complexed forms in vitro. Upon addition of LPS, the intensity of the main band decreased in a dose-dependent manner, while bands of higher molecular weight appeared, suggesting that RBD interacts with LPS, leading to the formation of larger complexes and/or conformational rearrangements. For the XBB.1.5 RBD, the main band was detected between 146 and 242 kDa, and was accompanied by several additional discrete bands, indicating that under native conditions the protein also exists in multiple oligomeric states. In the presence of LPS, the intensity of the main band completely disappeared, while new bands of higher molecular weight appeared in a dose-dependent manner, again consistent with the formation of larger complexes or structural rearrangements upon LPS binding. For the BA.1 and BA.2.86 RBDs, the main signal was detected at the top of the gel, suggesting that both proteins form large native aggregates that do not readily migrate, or that their conformation is unfavorable for Coomassie binding, resulting in insufficient negative charge for electrophoretic mobility. We therefore interpret these species as large native assemblies formed under experimental conditions, without inferring that they directly represent defined physiological oligomers in vivo. Importantly, upon addition of LPS, both BA.1 and BA.2.86 exhibited a clear, dose-dependent redistribution of signal toward distinct migrating species, indicating that LPS binding alters their native assembly state. Across all variants, the concentration-dependent migration shifts support a specific interaction with LPS rather than nonspecific aggregation. While BN-PAGE does not allow precise determination of structural organization in vivo, these data provide qualitative evidence that LPS binding promotes formation of higher-order RBD-containing complexes and/or conformational rearrangements under native conditions.

To understand the underlying molecular mechanisms of interaction, we performed atomic-resolution molecular dynamics (MD) simulations of the RBD of XBB.1.5 and BA.2.86 variants in the presence of lipid A, similarly to our previous study for the Wuhan-Hu-1 and BA.1 strains [12]. In the simulations, a lipid A molecule was placed approximately 2 nm away from the cryptic pocket on the RBD and 3 independent, unbiased MD simulations were performed for each variant. In all simulations, we found that lipid A spontaneously bound to the cryptic hydrophobic pocket within the first 100 ns and remained stably bound throughout the rest of the simulations, as shown by the overlaid snapshots (Fig. S5A) and root mean square deviation (RMSD) of the lipid (Fig. S5B). Cluster analysis shows that the key hydrophobic residues that interact with the lipid are similar to those in the Wuhan-Hu-1 and Omicron BA.1 variants (Fig. 2B). Multiple sequence alignment indicates that these hydrophobic residues in the RBD are universally conserved across all former VOCs as well as the XBB.1.5 and BA.2.86 variants (Fig. S6), suggesting that LPS binding persists throughout viral evolution.

Importantly, we observed a similar structural distortion of the gating helix region (depicted in blue in Fig. 2B) as previously reported for the Omicron BA.1 S protein [12]. This is not surprising given that the XBB.1.5 and BA.2.86 variants also have 3 serine residues mutated to nonpolar residues in this region similar to BA.1, with an additional threonine-to-alanine mutation nearby. Simulations of the ancestral Wuhan-Hu-1 RBD bound to lipid A shows that the serine residues form hydrogen bonds with the β-1,6′-linked glucosamine moieties of the lipid headgroup [12]. Mutations of these serine residues and the adjacent threonine would therefore result in a loss of interactions, leading to structural distortion of the gating helix.

### Lipid A binding affinity attenuated in emerging variants

**Fig. 3.**
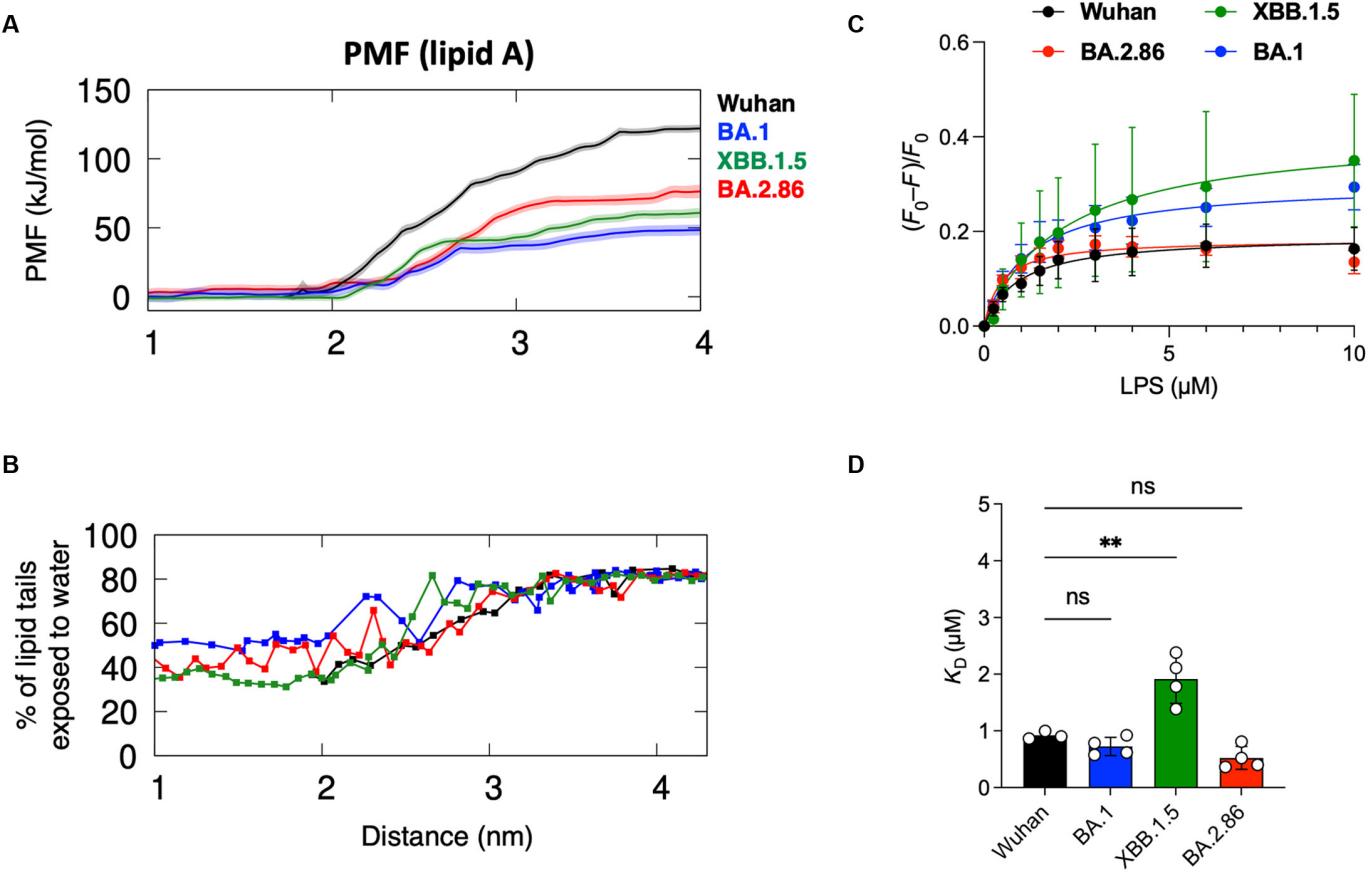
LPS binding affinities to S protein RBD from emerging variants. (A) Steered MD was performed to extract the lipid A molecule from the binding site, and umbrella sampling simulations were conducted to determine the PMF for (un)binding. The graph compares PMFs for Omicron BA.2.86 (red), Omicron XBB.1.5 (green), Wuhan-Hu-1 (black), and Omicron BA.1 (blue). Shaded areas show standard deviations estimated using bootstrapping. (B) Percentage of lipid tails exposed to water in each umbrella sampling window. Cutoff distance for water exposure is 0.4 nm. The average values from the converged portion of the simulations are shown. (C) The binding affinities of *E. coli* LPS to SARS-CoV-2 S protein RBDs from different variants were investigated by measuring intrinsic fluorescence. The extent of intrinsic fluorescence quenching [(*F*_0_ − *F*)/*F*_0_] of SARS-CoV-2 S protein RBD and its mutants is shown as a function of LPS concentration. *F*_0_ and *F* are the fluorescence intensities at 337 nm in the absence and in the presence of LPS. (D) The histograms show the *K*_D_ constants obtained from the curves in (C). Data are shown as mean ± SD (*n* = 3 to 4). *P* values were determined using a one-way ANOVA with Dunnett posttest. **P < 0.01; ns, not significant.

Due to the structural distortion at the RBD pocket gating helix, we hypothesized that lipid A binding in XBB.1.5 and BA.2.86 should be weaker compared to Wuhan-Hu-1 [12]. To test this hypothesis, we performed steered MD simulations to extract lipid A from the RBD of XBB.1.5 and BA.2.86, followed by umbrella sampling simulations to estimate the potential of mean force (PMF) values along the dissociation pathway. Indeed, our PMF calculations suggest that lipid A binds with lower affinities to both XBB.1.5 and BA.2.86 RBD with a free energy estimate of around 60 and 75 kJ mol^−1^, respectively (Fig. 3A). These values are substantially lower than the ancestral Wuhan-Hu-1 variant at around 125 kJ mol^−1^, and closer to the BA.1 variant at around 50 kJ mol^−1^. The percentages of lipid tail exposure to solvent when lipid A is close to the RBD were similar to that of Wuhan-Hu-1; however, as the lipid exits the cavity, it matches that of the BA.1 variant, showing higher overall exposure to water, likely due to unfolding of the gating helix (Fig. 3B). Collectively, our PMF calculations for lipid A binding to the RBD of recent VOIs predict a reduction in lipid A binding affinity over time.

### Full-length LPS maintains high binding affinities in most emerging variants

To validate our in silico prediction of the reduced binding affinities in emerging variants, we performed fluorescence quenching experiments. We found that higher doses of LPS were required to attain the same level of quenching of intrinsic fluorescence in the XBB.1.5 RBD compared to the ancestral Wuhan-Hu-1 RBD (Fig. 3C, left), suggesting a lower binding affinity in this variant, which agrees with our PMF data of lipid A binding. However, similar doses of LPS resulted in quenching of fluorescence in the BA.1 and BA.2.86 RBDs compared to the Wuhan-Hu-1 RBD, denoting similar binding affinities (Fig. 3C, right). The *K*_D_ values of LPS binding obtained from these fluorescence data for the Wuhan-Hu-1, BA.1, and BA.2.86 variants are around 1 μM, with no significant difference between them (Fig. 3D). In contrast, the *K*_D_ value for the XBB.1.5 variant is around 2 μM, indicating weaker LPS binding.

The discrepancies of LPS binding affinities compared to the PMF calculation of lipid A binding were surprising. We speculate that the binding affinity of the full-length LPS is dependent upon 2 factors: (a) lipid tails (the primary component of lipid A) and (b) the large extended sugar headgroups. Our PMF calculations predicted that the RBDs of all emerging variants have weaker lipid A binding affinities than the ancestral Wuhan-Hu-1 variant due to mutations nearby the cryptic pocket. However, this prediction only captures the lipid tail component of LPS, but not the sugar headgroups.

### Increase in positive charge on the surface of RBD could affect LPS binding

We next hypothesized that this inconsistent trend of binding affinities between lipid A and LPS could arise from the interaction of sugar moieties outside the lipid A moiety of LPS with the surface of the RBD on the exterior of the cryptic pocket. *E. coli* LPS is made of a hexa-acylated lipid A molecule connected to a series of sugar molecules, i.e., core oligosaccharides, which consist of keto-deoxyoctulosonate sugars, heptoses, and hexoses, as well as the O-antigen, which comprises highly variable, repetitive polysaccharides (Fig. S7). The R1 type core oligosaccharide of *E. coli* is made of a conserved inner core containing 2 phosphorylated mannoheptoses. These additional phosphate groups increase the overall charge of LPS to −10 *e* compared to −4 *e* for lipid A. The long strings of sugars with extra acidic groups in a full-length LPS molecule will likely interact with the surface of RBD; therefore, changes to the electrostatic properties of the RBD surface could affect binding affinities.

**Fig. 4.**
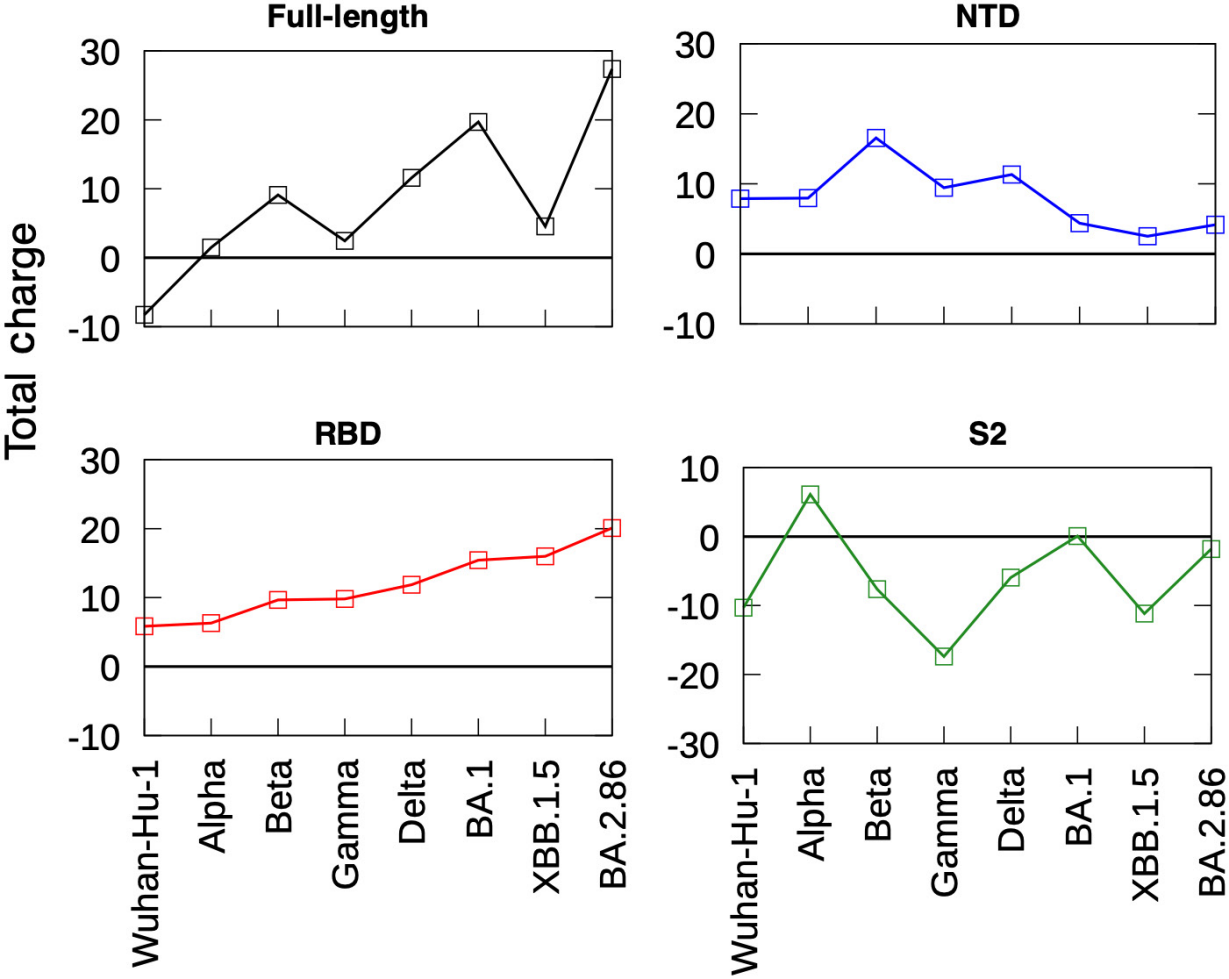
Comparison of S protein charge between SARS-CoV-2 variants. The total charge at pH 7 for S protein from various variants arranged chronologically. p*K*_a_ (where *K*_a_ is the acid dissociation constant) calculation was carried out using PROPKA on full-length S protein (black), NTD (blue), RBD (red), and S2 subunit (green).

To explore the surface charges of the S protein over time, we mapped the electrostatic surface potentials of the emerging variants and compared them to the ancestral variant (Fig. S8). We found additional positively charged patches on the surface of the S protein, particularly around the S1 subunit, in emerging variants. To quantify this result, we calculated the total surface charge of the full-length S protein trimer from various emerging variants in chronological order (Fig. 4). Intriguingly, the trend of increasing total positive charge on the full-length S protein over time does not prevail in all variants when arranged chronologically. For example, the Gamma and XBB.1.5 variants have lower total positive charges than the Beta and BA.1 variants, respectively, that emerged before them.

Delineating the surface charge to separate domains of the S protein, however, revealed an upward trend in the net positive charge on the surface of the RBD. At pH 7, the ancestral Wuhan-Hu-1 RBD has an estimated charge of +6 *e*, which increases to +15 *e* in Omicron BA.1 and +20 *e* in the BA.2.86 variant. In contrast, there is no clear trend in surface charge changes in the NTD and S2 subunits over the course of virus evolution. Thus, the accumulation of positive charge is concentrated on the RBD. It is possible that this continuous increase in net positive charge of the RBD surface favors the binding of sugar moieties in LPS. As the mutations around the RBD cryptic pocket depicted in Fig. 1 could aggravate lipid A binding, the increase in net positive charge on the RBD surface could compensate for this via more favorable sugar binding, resulting in a similar binding affinity of the overall LPS molecule.

Interestingly, the LPS binding affinity for RBD of the XBB.1.5 variant is lower compared to Wuhan-Hu-1 and the other emerging variants. We note that there is a small increase in surface charge over time between the RBD of BA.1 and XBB.1.5 (+15.4 *e* and +15.9 *e*, respectively). It is possible that this small increase in surface charge is not enough to compensate for the reduced lipid A binding affinity, resulting in the weaker overall LPS binding. We repeated our intrinsic fluorescence quenching experiment using lipid A for the Wuhan-Hu-1 and XBB.1.5 variants (Fig. S9). Consistent with the results from our LPS experiment shown in Fig. 3C, higher doses of lipid A were required to achieve the same level of quenching in XBB.1.5 compared to Wuhan-Hu-1, suggesting a lower lipid A binding affinity to the former, which agrees with our PMF calculations. We hypothesize that the mutations around the cryptic binding pocket reduce lipid A binding affinity, while the small changes in surface charge compared to the previous variants are inadequate to increase the overall LPS binding affinity. However, further experimental validation, such as reverse mutagenesis to restore the Wuhan-Hu-1 residues around the gating helix of XBB.1.5, would be valuable to test this hypothesis.

### The binding of LPS sugar moieties to RBD surface

**Fig. 5.**
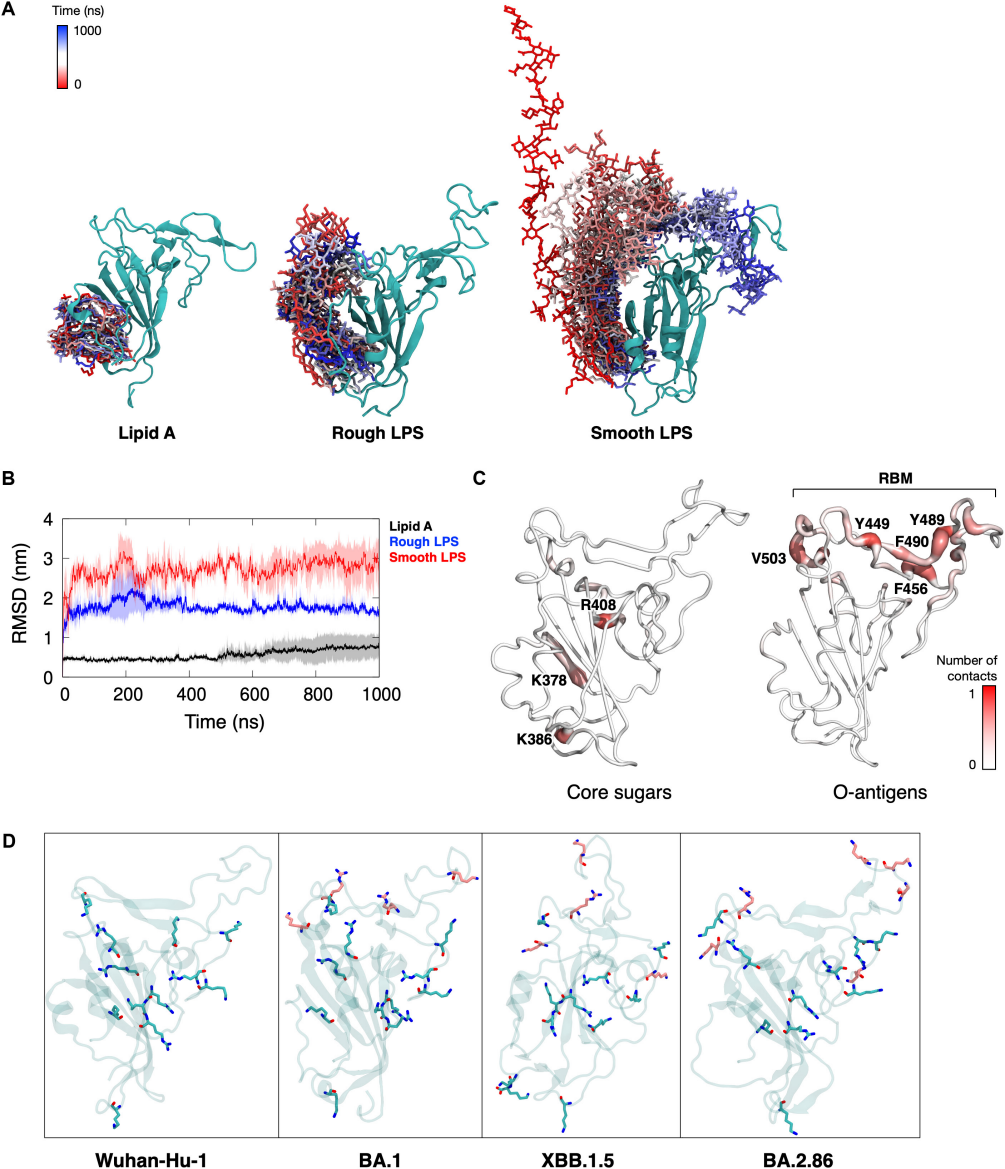
Simulation of full-length *E. coli* LPS with spike RBD. (A) Overlaid snapshots taken every 100 ns from a 1-μs simulations of RBD from the Wuhan-Hu-1 variant bound to lipid A (left), rough LPS (middle), and smooth LPS (right). The lipids are colored from red (0 ns) to blue (1,000 ns), while the protein is shown in cyan. (B) Average RMSD of lipid A, rough LPS, and smooth LPS, after least squares fit to the structure of the RBD–lipid complex. The thick lines show the average values from 3 independent simulations, whereas the shaded areas show standard deviations. (C) Contact analysis was performed on the simulation of RBD–smooth LPS. The figure shows average number of contacts made for each residue in the protein with the core sugars (left), and with the O-antigens (right) mapped to the structure of the Wuhan-Hu-1 RBD. Residues that made significant contacts are labeled. The position of the receptor binding motif (RBM) is also labeled. (D) Basic residues (arginine and lysine) found on the surface of the RBD. Residues that are present on the ancestral Wuhan-Hu-1 variant are colored cyan, while additional residues that are present only in emerging variants are colored pink.

To elucidate the binding of full-length *E. coli* LPS to the RBD, we performed atomic-resolution simulations of the RBD from the ancestral Wuhan-Hu-1 variant bound to rough and smooth LPS molecules. We employed an LPS structural model with O-antigens from *E. coli* O111, which was the strain used in our experiments. We found that while the lipid A component binds stably to the hydrophobic pocket, the sugar moieties were more flexible throughout the simulations (Fig. 5A). The sugar molecules can bend toward the RBD and bind to its surface. The large motion of the sugar molecules resulted in higher overall RMSD values for the LPS molecules compared to our previous simulations with lipid A (Fig. 5B).

We performed contact analysis to determine which residues on the RBD interacted with the sugar moieties (Fig. 5C). The core sugars interacted with a group of positively charged residues nearby the hydrophobic pocket (K378, K386, and R408). The inner core sugars have 2 negatively charged phosphate groups (Fig. S7B), which explains the preferential binding to basic residues. The O-antigens interacted with residues that are further away from the lipid A binding pocket, forming intermittent hydrogen bonds with residues Y449, Y489, and F490, which are part of the RBM. To enhance the sampling made by these sugar moieties, we performed additional simulations of RBD bound to smooth LPS at a higher temperature of 500 K. To preserve the secondary structure of the protein at this high temperature, we applied positional restraints to the backbone atoms (more details in Materials and Methods). Similarly, we found that the inner core phosphorylated sugars formed salt bridges with basic residues around the lipid A binding pocket (K378 and R408), while the O-antigens sampled a much larger conformational space that allows for distal interactions including with the RBM (Fig. S10A and B). Importantly, the salt bridges between the phosphate groups of the LPS inner core and the positively charged residues on the RBD surface represent the most prominent interactions throughout the simulations (Fig. S10C and D). While the Wuhan-Hu-1 RBD contains several positively charged residues that can interact with the LPS inner core sugar moieties, such as K378 and R408, the increasing trend of positive surface charge in emerging variants indicates that SARS-CoV-2 evolution over time gains additional basic residues on the surface of RBD. Indeed, we found up to 6 extra basic residues (arginine/lysine) on the surface of emerging variants compared to Wuhan-Hu-1 (Fig. 5D). We speculate that due to the highly mobile nature of the string of sugar moieties (Fig. 5A and Fig. S10A), having more positively charged residues to interact with would likely increase the binding affinity of the LPS headgroup. Collectively, our simulations of RBD bound to full-length LPS molecules corroborate the hypothesis that more favorable RBD interactions with the LPS sugar moieties could compensate for the reduced affinity of the lipid A component.

## Discussion

Various clinical evidence has demonstrated that Gram-negative bacterial LPS is pivotal in severe COVID-19 illnesses and even death [25–29]. To explore the underlying molecular mechanism linking LPS and COVID-19, we have previously shown that the SARS-CoV-2 S protein acts as an additional LPS chaperone that transfers the lipid to its downstream receptors, which can result in hyperinflammation [11,12,30]. Here, we analyzed the effect of mutations in the S protein of emerging variants over the course of the pandemic upon LPS interaction. Up to 4 mutations were found nearby the gating helix of the RBD cryptic pocket, which represents the high-affinity LPS binding site. Our PMF calculations indicate that these mutations result in a reduced lipid A binding affinity to S protein RBD from Omicron BA.1, XBB.1.5, and BA.2.86 compared to the ancestral Wuhan-Hu-1 variant. Intriguingly, fluorescence quenching experiments performed using full-length *E. coli* LPS depicted no significant changes to the *K*_D_ values for BA.1 and BA.2.86 variants, which suggests a potential compensatory mechanism by the sugar moieties. There is a steady increase in positive charge on the surface of the RBD over time, which could favor binding of anionic LPS sugar molecules. Collectively, our study demonstrates how emerging variants maintain the LPS binding capacity despite harboring numerous mutations via a fine balance of interactions involving both lipid tails and the long sugar chain.

As SARS-CoV-2 evolved over the years, the S protein has accumulated positive charges on the surface, as demonstrated by the recent emerging variants having a higher net total positive charge compared to previous variants [31]. In this study, we showed that this increase in positive charge is clustered around the RBD, highlighting the importance of these changes in S protein–receptor binding. Indeed, binding affinity measurements indicate stronger affinity for the BA.2.86 RBD to the angiotensin converting enzyme 2 (ACE2) compared to previous variants [32–34]. SARS-CoV-2 infection is also dependent on host heparan sulfate, whereby initial binding of the S protein to heparan sulfate on the cell surface enhances recognition of ACE2 [35]. The trend of increasing positive charge on the S protein of emerging variants improves binding to the negatively charged cellular heparan sulfate [36]. Similarly, our study suggests that these positively charged surfaces on the RBD improve binding of LPS core sugars and O-antigens, counterbalancing the negative effects of mutations around the lipid A binding pocket. As the compensatory role of sugar moieties in our study is primarily inferred from structural analysis and MD simulations, further experimental validation—such as reverse mutagenesis of residues around the lipid A binding pocket to the equivalent ones in the ancestral Wuhan-Hu-1 S protein to restore lipid A binding affinity—would be desirable to corroborate this hypothesis.

The S protein RBD is a hotspot for mutations due to its role in antibody binding. As such, it is not surprising that some mutations occur in close proximity to the cryptic hydrophobic pocket. We therefore focused our study on this particular LPS binding site. However, it is worth noting that LPS is also capable of binding to another cryptic pocket on the NTD, as well as an inter-protomeric surface groove in the S2 subunit [12,37], which are not covered by our current study. While there are no mutations found near these 2 binding sites, other mutations could still affect LPS binding via an allosteric network [37]. Previous studies have shown that the binding of haem metabolites to the NTD cryptic pocket disrupts the architecture of distal antibody epitopes [15,38]. Therefore, it is possible for the mutations at the NTD antibody epitopes to control the opening of the cryptic pocket, hence affecting LPS binding. Indeed, a structure-based statistical mechanical model of allostery identified residues 205 to 209 at the opening of the NTD pocket as a hotspot for allosteric communications [39]. Our previous study showed that full-length S protein and the S1 subunit of Omicron BA.1 have lower affinity for LPS compared to the Wuhan-Hu-1 counterparts [12]. As our current study demonstrated similar binding affinities to the RBD for these 2 variants, it is likely that the weakened LPS binding to the full-length S protein and the S1 subunit of BA.1 variant are due to the mutations in the NTD. However, the specific nature of the allosteric communication between these mutations and the NTD cryptic pocket is likely to be complex and would require further investigation.

From an evolutionary perspective, the emergence of lipid-binding pockets on a viral surface protein is intriguing. Our previous sequence analysis of beta-coronaviruses revealed that LPS binding residues are conserved across different species [12], while our native gel electrophoresis showed that the SARS-CoV S protein indeed also interacts with LPS [11]. This evidence suggests that the ability to bind LPS is not unique to SARS-CoV-2 but rather a universal feature among many coronaviruses. Interestingly, during the evolution of SARS-CoV-2, key hydrophobic residues for LPS binding are also preserved despite numerous mutations arising in emerging variants (Fig. S6). While the reason why these viruses evolve to bind LPS remains poorly understood, one possible explanation is to promote viral spread through bacteria–virus interactions as has been documented for poliovirus [40] and influenza virus [41]. Indeed, the rate of bacterial co-infection among COVID-19 patients can reach as high as 23.5% for intensive care unit (ICU) patients [42]. The evolution of SARS-CoV-2 has improved the S protein RBD binding affinity to the host ACE2 receptor and cell surface glycosaminoglycans via increased positive charge [32–34,36]. It is thus interesting that the virus uses the same mechanism to retain LPS binding throughout evolution, hence potentially maintaining the ability for bacteria–virus coinfection.

The physiological relevance of our findings stems from the high local LPS concentration in clinical settings. Longitudinal patient data demonstrate increasing circulating LPS levels in COVID-19 nonsurvivors compared with survivors [29], while cohort studies using validated proxies of endotoxin exposure (such as LPS binding protein and soluble CD14) link microbial translocation signatures to increased mortality [43]. Although endotoxin activity assay studies report variable mortality associations, they consistently indicate that endotoxemia is common in severe COVID-19. High local endotoxin levels have also been reported in inflamed tissue compartments, such as bronchoalveolar lavage fluid during Gram-negative pneumonia, where LPS concentration can reach tens to hundreds of EU/ml [44]. While we showed that LPS binds with micromolar affinity to an isolated S protein RBD, it is important to note that the S protein forms a trimer and each protomer has multiple LPS binding sites. Hence, multivalent binding of LPS to S protein may have cooperative effects. Indeed, our previous study revealed that LPS binds to the full-length Wuhan-Hu-1 S protein with a *K*_D_ of ~40 nM [11], similar to CD14. Consistent with this framework, S protein has been shown to synergize with very low LPS concentrations to amplify nuclear factor κB (NF-κB) activation in vitro and in vivo [11,12]. Moreover, S protein-mediated modulation of LPS administrated to the lungs can markedly boost inflammation in vivo in an experimental mouse model [30], further supporting the plausibility of this mechanism under clinically relevant inflammatory conditions.

While our study focused on the observed molecular interaction between the S protein RBD and LPS, we acknowledge that the translation to the in vivo environment and possible implications on hyperinflammation remain a complex issue that would require further studies. The S protein is cleaved by furin protease into S1 and S2 subunits. While the S1 subunit is shed away into circulation, it remains unclear whether a dissociated, independent RBD circulates systematically, as were used in the simulations and experiments in this study. The reduction of complexity to an isolated RBD in this study was necessary here to allow for the extensive free energy calculations across many variants and large-scale simulations with full-length LPS, which would otherwise be prohibitively expensive if the S protein trimer or even the S1 subunit was used. Nevertheless, our study provides important molecular insights into the S protein–LPS interactions across various SARS-CoV-2 variants.

In conclusion, despite numerous mutations, SARS-CoV-2 S protein RBD from emerging variants preserves its ability to bind to Gram-negative bacterial LPS. Our present study focused on 3 major Omicron variants (BA.1, XBB.1.5, and BA.2.86) from the years 2021 to 2023, which have since been displaced by the newer VOI, JN.1, and its subvariants, such as XEC, LP.8.1, XFG, and NB.1.8.1. Nevertheless, all hydrophobic residues predicted to interact with the LPS lipid tails are conserved in these newer variants, while an additional H445R mutation is present in some variants [45] that may increase the positive surface charge of RBD, in agreement with the trend reported here. This suggests that LPS binding is likely to remain conserved in the most recent SARS-CoV-2 variants that are circulating in 2026. S–LPS interaction drives hyperinflammation, which could manifest as severe complications such as acute respiratory distress syndrome (ARDS) and sepsis observed in COVID-19 patients at the beginning of the pandemic [19,46], as well as amyloid formation leading to neurological disorders [21]. Hence, even in newer variants, LPS binding remains a concern for causing severe COVID-19 symptoms, underscoring the importance of continued surveillance of the evolution of the SARS-CoV-2 virus.

## Materials and Methods

### Proteins

SARS-CoV-2 S RBDs from Wuhan-Hu-1 and Omicron variants XBB.1.5, BA.2.86, and BA.1.1 were purchased from ACROBiosystems (USA) in lyophilized form. The sequence of SARS-CoV-2 S RBD from Wuhan-Hu-1 contains AA Arg^319^–Lys^537^ (accession no. QHD43416.1); the sequence of SARS-CoV-2 S RBD Omicron variant XBB.1.5 contains AA Arg^319^–Lys^537^ [accession no. QHD43416.1 (G339H, R346T, L368I, S371F, S373P, S375F, T376A, D405N, R408S, K417N, N440K, V445P, G446S, N460K, S477N, T478K, E484A, F486P, F490S, Q498R, N501Y, Y505H)]; the sequence of SARS-CoV-2 S RBD Omicron variant BA.2.86 contains AA Arg^319^–Lys^537^ [accession no. QHD43416.1 (I332V, G339H, K356T, S371F, S373P, S375F, T376A, R403K, D405N, R408S, K417N, N440K, V445H, G446S, N450D, L452W, N460K, S477N, T478K, N481K, V483del, E484K, F486P, Q498R, N501Y, Y505H)]; the sequence of SARS-CoV-2 S RBD Omicron variant BA.1.1 contains AA Arg^319^–Lys^537^ [accession no. QHD43416.1 (G339D, R346K, S371L, S373P, S375F, K417N, N440K, G446S, S477N, T478K, E484A, Q493R, G496S, Q498R, N501Y, Y505H)]. All lyophilized proteins were reconstituted in endotoxin free water, aliquoted, and stored at −80 °C according to the manufacturer’s protocol. The purity was >95% for SARS-CoV-2 S RBDs from Wuhan-Hu-1 and Omicron variants XBB.1.5 and BA.1.1, and >90% for SARS-CoV-2 S RBD from Omicron variant BA.2.86.

### Multiple sequence alignment

Amino acid sequences of the S proteins from SARS-CoV-2 Wuhan-Hu-1, Alpha (B.1.1.7), Beta (B.1.351), Gamma (P.1), Delta (B.1.617.2), Omicron BA.1, Omicron XBB.1.5, and Omicron BA.2.86 were obtained from https://covariants.org, enabled by data from the GISAID consortium [22]. A multiple sequence alignment was generated using Jalview [47] and the ProbCons algorithm [48]. The positions of mutated residues were mapped to the cryo-EM structures of selected S protein ECDs using VMD [48]. The structures of S protein ECDs were obtained from the PDB (BA.1, PDB: 7T9K [49]; XBB.1.5, PDB: 8JYK [50]; BA.2.86, PDB: 8XLV [51]).

### BN-PAGE and Western blotting

S protein RBDs from Wuhan-Hu-1 and Omicron variants XBB.1.5, BA.2.86, and BA.1 (2 μg) were incubated with 100, 250, and 500 μg/ml *E. coli* LPS (Sigma-Aldrich, catalog no. L3024) for 30 min at 37 °C in a final volume of 20 μl. After incubation, samples were mixed with loading buffer, and 1 μg of each protein was loaded onto a BN-PAGE (Native PAGE BisTris Gel System 4%–16%, Invitrogen) and run at 150 V for 120 min. Proteins were transferred from the gel to a polyvinylidene difluoride (PVDF) membrane using the Trans-Blot Turbo (Bio-Rad). The membrane was blocked with 5% milk in phosphate-buffered saline with Tween 20 (PBS-T) for 1 h at room temperature. The membrane was then incubated with primary antibodies against the His-tag (1:2,000, Invitrogen, catalog no. MA1-21315) at 4 °C overnight, followed by incubation with horseradish peroxidase (HRP)-conjugated secondary antibody (1:2,000, Dako, catalog no. P0260) for 1 h at room temperature. Proteins were visualized by incubating the membrane with SuperSignal West Pico Chemiluminescent Substrate (Thermo Scientific) for 2 min, followed by detection using a ChemiDoc XRS Imager (Bio-Rad).

### Unbiased all-atom MD simulations

To investigate the binding of lipid A with S protein RBD from the XBB.1.5 and BA.2.86 variants, we performed all-atom MD simulations. The structure of SARS-CoV-2 XBB.1.5 S protein RBD was extracted from the cryo-EM structures of its ECD (PDB: 8JYK [50]). The structure of SARS-CoV-2 BA.2.86 S protein RBD was extracted from the cryo-EM structure of the RBD complexed with ACE2 (PDB: 8QSQ [33]). The structure of *E. coli* lipid A molecule was obtained from CHARMM-GUI LPS modeler [52]. To set up the system, a lipid A molecule was placed approximately 2 nm from the cryptic hydrophobic pocket of the RBD. N-glycans were added to the putative N-glycosylation site, N343, using the CHARMM-GUI glycan readers and modelers [53]. The most dominant isoform was added based on mass spectrometry data [54]. The K356T mutation in BA.2.86 resulted in an additional putative N-glycosylation site at residue N354. As this residue has a similar surface accessibility as the nearby, N343, we added a similar hybrid glycan to the N354 residue. Both protein and lipid were parameterised using the all-atom CHARMM36m forcefield [55]. The protein and lipid were placed in a 10 nm × 10 nm × 10 nm box, which was solvated with TIP3P water molecules and 0.15 M NaCl salt. Steepest descent energy minimisation was performed to remove any inter-atomic clashes. A short 125-ps equilibration simulation was conducted following the standard CHARMM-GUI protocols [56]. Positional restraints with a force constant of 1,000 kJ mol^−1^ nm^−1^ were applied to the backbone atoms of the protein and the heavy atoms of the lipids. For production simulations, the positional restraints were removed and 3 independent 1-μs simulations were run starting from different initial velocity distributions. The temperature of the system was maintained at 310 K using the Nosé–Hoover thermostat [57,58], while an isotropic coupling to a Parrinello–Rahman barostat was used to maintain the pressure at 1 atm [59]. The electrostatic interactions were computed using the smooth particle mesh Ewald (PME) method [60] with a real space cutoff of 1.2 nm. The van der Waals interactions were truncated at 1.2 nm with a force-switch smoothing function applied between 1.0 and 1.2 nm. Constraints were applied on all covalent bonds involving hydrogen atoms using the LINCS algorithm [61], and an integration time step of 2 fs was used. All simulations were performed using GROMACS 2021 [62]. To determine the representative structure of the protein–lipid A complex, we conducted a clustering analysis using concatenated trajectories from the 3 production simulations. The GROMOS algorithm was used with an RMSD cutoff of 0.35 nm [63].

To understand the role of sugar moieties in LPS binding, we performed simulations of Wuhan-Hu-1 RBD S protein (extracted from our full-length S glycoprotein trimer model [16]) with *E. coli* rough and smooth LPS molecules. The LPS molecules were modeled using CHARMM-GUI LPS modeler [52]. For the smooth LPS molecule, the O-antigens used were that from *E. coli* O111 species to match the strain used in our experimental procedures. A rough or smooth LPS molecule was placed approximately 2 nm from the cryptic pocket of the RBD inside 10 × 10 × 10 nm^3^ or 15 × 15 × 15 nm^3^ boxes, respectively, and solvated with TIP3P water molecules and 0.15 M NaCl salt. Energy minimisation, equilibration, and production simulations followed the same protocols mentioned above for lipid A. Additionally, we performed 3 independent 500-ns simulations at 500 K for the RBD–smooth LPS complex to improve the sampling of the sugar moieties. For these simulations, positional restraints with a force constant of 500 kJ mol^−1^ nm^−1^ were applied to the backbone atoms of the protein.

### Umbrella sampling simulations

To accurately quantify the binding energies of lipid A to the BA.2.86 and XBB.1.5 S protein RBD, we calculated the PMF using a series of umbrella sampling MD simulations. The central structures of the top cluster obtained from clustering analysis of unbiased MD simulations were used as the starting structure. Firstly, a steered MD simulation was performed along a reaction coordinate parallel to the lipid dissociation pathway from the protein. Lipid A was pulled away from the binding pocket at a constant velocity of 0.1 nm ns^−1^ using an elastic spring with a force constant of 1,000 kJ mol^−1^ nm^−2^ applied to its center of mass. The protein was maintained at its original position by applying positional restraints on the backbone atoms with a force constant of 1,000 kJ mol^−1^ nm^−2^. An initial set of 50 frames was selected from this trajectory for subsequent umbrella sampling simulations based on the distance between the centers of mass of the lipid and the protein and a separation of 0.1 nm between adjacent frames. For each umbrella sampling window, a 100-ns simulation was performed whereby the center of mass of the lipid is restrained in the vector of the reaction coordinate by a force constant of 1,000 kJ mol^−1^ nm^−2^, while no restraints were applied to the protein. To compute the PMF, the GROMACS *gmx wham* based on weighted histogram analysis method (WHAM) was used [64]. To ensure adequate sampling along the reaction coordinate, histogram overlap was plotted (Fig. S11A); additional simulations were subsequently run to fill in any areas that had been poorly sampled. To estimate the statistical error, 100 bootstrap trials were performed for each PMF calculation. To ensure convergence was achieved within the timescale of the simulations, we generated PMF profiles using increasing amounts of simulation sampling time (Fig. S11B).

### Quenching of intrinsic fluorescence

S protein RBDs from Wuhan-Hu-1 and Omicron variants XBB.1.5, BA.2.86, and BA.1 (200 μl at 2 μM, PBS, pH 7.4) were used to measure the intrinsic fluorescence after titration with increasing concentrations of LPS (0 to 4 μM). Emission fluorescence spectra were recorded between 300 and 450 nm following excitation at 280 nm, using a Jasco J-810 spectropolarimeter equipped with an FMO-427S fluorescence module. The scan rate was set to 200 nm/min, and the slit width was 2 nm. Fluorescence quenching and *K*_D_ were determined by nonlinear regression analysis of the full dose–response curves according to [12,65]. This fitting approach accounts for differences in maximal quenching and overall signal amplitude across variants. Results are shown as mean values ± SD from 3 or 4 measurements.

In another set of experiments, S protein RBDs from Wuhan-Hu-1 and Omicron variant XBB.1.5 (200 μl at 2 μM, PBS, pH 7.4) were mixed with *E. coli* lipid A (0 to 34.3 μM). The experiments were performed, and the data were analyzed as described above.

### Surface charge calculation

To calculate the surface charge of the full-length S protein and its domains, we built full-length models of the S protein from the Alpha, Beta, Gamma, Delta, BA.1, XBB.1.5, and BA.2.86 variants using integrative modeling technique via Modeller version 10.5 [66]. The cryo-EM structures of S protein from the respective variants were used as the templates for the ECD: Alpha, 7LWT [67]; Beta, 7N1Q [68]; Gamma, 7SBS [68]; Delta, 7SBL [68]; BA.1, 7T9J and 7T9K [49]; XBB.1.5, 8JYK [50]; BA.2.86, 8XLV [51]). The heptad repeat 2 and transmembrane (TM) domains were modeled based on the nuclear magnetic resonance (NMR) structure of SARS-CoV HR2 domain (PDB: 2FXP [69]) and the NMR structure of human immunodeficiency virus 1 (HIV-1) gp-41 TM domain (PDB: 5JYN [70]), respectively. These models were built following the same methodology as our previous study [16]. PROPKA version 3 was used to calculate the total surface charges of full-length models. To isolate the surface charges of individual domains, residues outside of these domains were converted to dummy atoms with zero charges. The results presented represent the expected surface charges of a folded protein at pH 7. The electrostatic surface potentials were calculated and mapped using PyMOL APBS plugin.

## Data Availability

Simulation data including S protein models for emerging variants, initial and final coordinates of simulations with lipid A and LPS, as well as simulation input files have been deposited to https://zenodo.org/records/17626946.
