# Supplementary material for: Conservation of Bacterial Lipopolysaccharide Binding by SARS-CoV-2 Spike across Major Viral Variants
Source: Comput Struct Biotechnol J. 2026 Apr 2;35(1):0040. doi: 10.34133/csbj.0040 (PMC13082671; doi:10.34133/csbj.0040)
Supplement: Supplementary 1 — Figs. S1 to S11 [file csbj.0040.f1.docx]

**Supplementary Figures**

**Supplementary Figure 1: Multiple sequence alignment of S proteins.**  MSA from SARS-CoV-2 Wuhan, Alpha, Beta, Gamma, Delta, BA.1, XBB.1.5, and BA.2.86 variants represented as fingerprints with conserved sequences coloured in dark grey. The positions of the NTD, RBD, and the S2 subunit are marked.

**Supplementary Figure 2: Mutations on the BA.1 variant S protein relative to LPS binding sites. (A)** The list of mutations on the S protein of SARS-CoV-2 BA.1 variant compared to the ancestral Wuhan variant. **(B)** The positions of the mutations mapped onto the structure of the protein with the LPS binding sites shown in dashed circles as depicted in Figure 1.

**Supplementary Figure 3: Mutations on the XBB.1.5 variant S protein relative to LPS binding sites. (A)** The list of mutations on the S protein of SARS-CoV-2 XBB.1.5 variant compared to the ancestral Wuhan variant. **(B)** The positions of the mutations mapped onto the structure of the protein with the LPS binding sites shown in dashed circles as depicted in Figure 1.

**Supplementary Figure 4:** **Evaluation of SARS-CoV-2 S protein RBD and its mutants by SDS-PAGE.** The purity of the lyophilized proteins after solubilization was assessed using SDS-PAGE followed by Coomassie staining. For each protein, 2 µg were loaded onto the gel and separated under reducing conditions.

**Supplementary Figure 5: Binding stability of lipid A to the RBD of XBB.1.5 and BA.2.86. (A)** Overlaid snapshots taken every 50 ns over the course of a 1,000 ns simulations showing the spontaneous binding of lipid A to the cryptic pocket of RBD from XBB.1.5 (left) and BA.2.86 variants (right). **(B)** Average RMSD of lipid A after least squares fit to its initial structure. Thick lines show the average values from three independent simulations, while the shaded regions depict the standard deviations.

**Supplementary Figure 6: Sequence alignment of SARS-CoV-2 spike RBD.** Sequence alignment of RBD from Wuhan-Hu-1 and emerging variants with the consensus sequence shown on top as a WebLogo representation. Identical residues are shown in blue and similar residues are shown in magenta. Green triangles depict residues that interact with LPS lipid tails, whereas the red circles highlight residues at the gating helix.

**Supplementary Figure 7: Structural comparison between E. coli lipid A, rough LPS and smooth LPS. (A)** The structures were generated using CHARMM-GUI LPS Modeller. For smooth LPS, the O-antigen represents *E. coli* O111. The total charge of each lipid is shown below. **(B)** The chemical structure of *E. coli* R1 LPS inner core sugars. Kdo, 3-deoxy-D-manno-oct-2-ulosonic acid; P-Hep, phosphorylated D-manno-heptose; Hep, L-glycero-D-manno-heptose. **(C)** The chemical structure of *E. coli* R1 LPS outer core sugars. Glc, D-glucose; Gal, D-galactose. **(D)** The chemical structure of *E. coli* O111 O-antigen. GlcNac, N-acetyl-D-glucosamine; Gal, D-galactose; Glc, D-glucose; Col, L-colitose.

**Supplementary Figure 8: Comparison of S protein electrostatic surface potential between SARS-CoV-2 variants.** **(A, B, C, and D)** The electrostatic surface potential of the full-length S protein trimer from the Wuhan-Hu-1, BA.1, XBB.1.5, and BA.2.86 variants, respectively, calculated using PyMOL APBS tool. Quantitative calculation of surface charge of the different domains of S protein trimer is shown in Figure 4.

**Supplementary Figure 9:** **Binding affinity of Lipid A to SARS-CoV-2 S protein RBD.** The binding affinity of *E. coli* Lipid A to SARS-CoV-2 S protein RBD and its Omicron variant XBB.1.5 was investigated by measuring intrinsic fluorescence. **(A)** The extent of intrinsic fluorescence quenching [(F0-F)/F0] of SARS-CoV-2 S protein RBD and its Omicron variant XBB.1.5 is shown as a function of Lipid A concentration. F0 and F are the fluorescence intensities at 337 nm in the absence and in the presence of Lipid A. **(B)** The histograms show the KD constants obtained from the curves in (A). Data are shown as mean ± SD (n=3). P value was determined using an unpaired t test with Welch's correction. *P < 0.05.

**Supplementary Figure 10: Enhanced sampling of LPS sugars using high-temperature simulations. (A)** Three independent 500 ns simulation was performed using Wuhan RBD bound to smooth LPS at 500 K. Positional restraints were applied to the backbone atoms of the protein. The figure shows overlaid snapshots taken every 10 ns for concatenated trajectories of all three simulations. The smooth LPS molecule was coloured based on its components: lipid A in black, core sugars in blue, and O-antigens in red. The RBD was shown in cyan. **(B)** Average number of contacts made for each residue in the protein with the core sugars (left) and with the O-antigens (right) mapped to the structure of Wuhan RBD. Residues that made significant contacts and the position of the RBM are labelled. **(C)** Average number of contacts made by top five residues with core sugars and O-antigens, with standard deviations between repeat simulations shown as error bars. **(D)** A snapshot at the end of one of the simulations showing a salt bridge formed between residue R408 and the phosphate group from LPS inner core sugar.

**Supplementary Figure 11: Sampling and convergence analyses for PMF calculations.** **(A)** Histogram overlap from all umbrella sampling windows of lipid A unbinding from the RBD of BA.2.86 and XBB.1.5. **(B)** PMF profiles generated with increasing lengths of simulation sampling. The PMF profiles converged after 70 ns and 60 ns for RBD of BA.2.86 and XBB.1.5, respectively.
